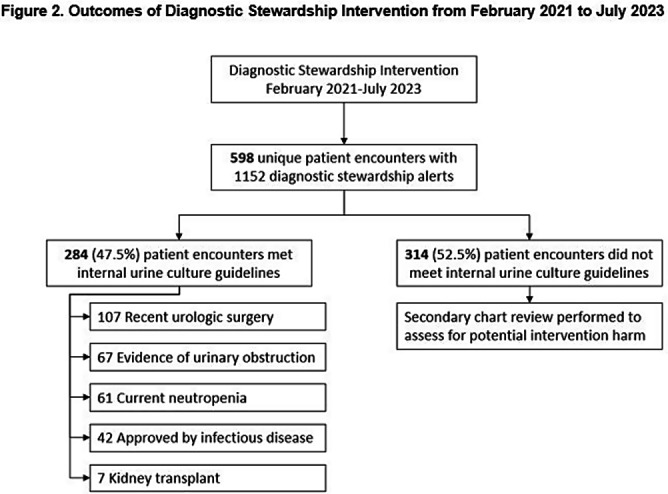# Impact and Safety of Diagnostic Stewardship to Improve Urine Culture Testing Among Patients with Indwelling Urinary Catheters

**DOI:** 10.1017/ash.2024.129

**Published:** 2024-09-16

**Authors:** Sarah Sansom, Audrey Goldstein, Michael Lin, Michael Schoeny, Ruth Kniuksta, Alexandra Seguin, Alexander Tomich, Brian Stein, John Segreti

**Affiliations:** Rush University Medical Center

## Abstract

**Background:** Indiscriminate urine culturing of patients with indwelling urinary catheters may lead to overdiagnosis of urinary tract infections, resulting in unnecessary antibiotic treatment and inaccurate reporting of catheter-associated urinary tract infections (CAUTIs) as a hospital quality metric. We evaluated the impact of a computerized diagnostic stewardship intervention to improve urine culture testing among patients with indwelling urinary catheters. **Methods:** We performed a single-center retrospective observational study at Rush University Medical Center from April 2018 – July 2023. In February 2021, we implemented a computerized clinical decision support tool to promote adherence to our internal urine culture guidelines for patients with indwelling urinary catheters. Providers were required to select one guideline criteria: 1) neutropenia, 2) kidney transplant, 3) recent urologic procedure, 4) urinary tract obstruction; or if none of the criteria were met, then an infectious diseases consultation was required for approval. We compared facility-wide CAUTI rate per 10,000 catheter days and standardized infection ratio (SIR) during baseline and intervention periods using ecologic models, controlling for time and for monthly Covid-19 hospitalizations. In the intervention period, we evaluated how providers responded to the intervention. Potential harm was defined as collection of a urine culture within 7 days of the intervention that resulted in a change in clinical management. **Results:** In unadjusted models, CAUTI rate decreased from 12.5 to 7.6 per 10,000 catheter days (p=0.04) and SIR decreased from 0.77 to 0.49 (p=0.09) during baseline vs intervention periods. In adjusted models, the CAUTI rate decreased from 6.9 to 5.5 per 10,000 catheter days (p=0.60) (Figure 1) and SIR decreased from 0.41 to 0.35 (p=0.65) during baseline vs intervention periods. Urine catheter standard utilization ratio (SUR) did not change (p=0.36). There were 598 patient encounters with ≥1 intervention. Selecting the first intervention for each encounter, 284 (47.5%) urine cultures met our guidelines for testing and 314 (52.5%) were averted (Figure 2). Of these, only 3 ( < 1 %) had a urine culture collected in the subsequent 7 days that resulted in change in clinical management. **Conclusion:** We observed a trend of decreased CAUTIs over time, but effect of our diagnostic stewardship intervention was difficult to assess due to healthcare disruption caused by Covid-19. Adverse outcomes were rare among patients who had a urine culture averted. A computerized clinical decision support tool may be safe and effective as part of a multimodal program to reduce unnecessary urine cultures in patients with indwelling urinary catheters.

**Figure:**
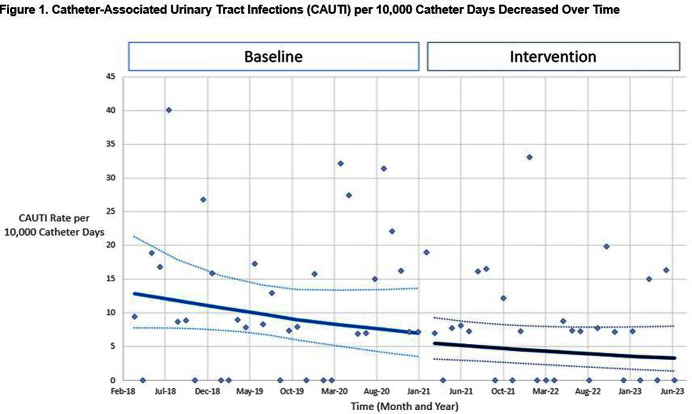

**Figure:**